# Urine-Derived Stem Cells for Epithelial Tissues Reconstruction and Wound Healing

**DOI:** 10.3390/pharmaceutics14081669

**Published:** 2022-08-11

**Authors:** Xiya Yin, Qingfeng Li, Patrick Michael McNutt, Yuanyuan Zhang

**Affiliations:** 1 Department of Burn and Plastic Surgery, West China Hospital, Sichuan University, Chengdu 610017, China; 2 Department of Plastic and Reconstructive Surgery, Shanghai Ninth People’s Hospital, Shanghai Jiao Tong University School of Medicine, Shanghai 200011, China; 3 Wake Forest Institute for Regenerative Medicine, Wake Forest University Health Sciences, Winston-Salem, NC 27101, USA

**Keywords:** urine-derived stem cell, skin, epidermal, urothelium, bioengineering, tissue regeneration, personalized medicine

## Abstract

Epithelial tissue injury can occur on any surface site of the body, particularly in the skin or urethral mucosa tissue, due to trauma, infection, inflammation, and toxic compounds. Both internal and external body epithelial tissue injuries can significantly affect patients’ quality of life, increase healthcare spending, and increase the global economic burden. Transplantation of epithelial tissue grafts is an effective treatment strategy in clinical settings. Autologous bio-engineered epithelia are common clinical skin substitutes that have the specific advantages of avoiding tissue rejection, obviating ethical concerns, reducing the risk of infection, and decreasing scarring compared to donor grafts. However, epithelial cells are often obtained from the individual’s skin and mucosa through invasive methods, which cause further injury or damage. Urine-derived stem cells (USC) of kidney origin, obtained via non-invasive acquisition, possess high stemness properties, self-renewal ability, trophic effects, multipotent differentiation potential, and immunomodulatory ability. These cells show versatile potential for tissue regeneration, with extensive evidence supporting their use in the repair of epidermal and urothelial injuries. We discuss the collection, isolation, culture, characterization, and differentiation of USC. We also discuss the use of USC for cellular therapies as well as the administration of USC-derived paracrine factors for epidermal and urothelial tissue repair. Specifically, we will discuss 3D constructions involving multiple types of USC-loaded hydrogels and USC-seeded scaffolds for use in cosmetic production testing, drug development, and disease modeling. In conclusion, urine-derived stem cells are a readily accessible autologous stem cell source well-suited for developing personalized medical treatments in epithelial tissue regeneration and drug testing.

## 1. Introduction

Epithelial tissues are stratified cell assemblies that cover the exterior surfaces of the body (i.e., skin), form internal passageways (i.e., mucosa in the urinary tract and gastrointestinal systems), line internal cavities, and form secretory glands [1]. Epithelia perform versatile critical functions such as protection, secretion, absorption, and immune defense. Under normal conditions, the epithelium is capable of rapid regeneration and self-repair through activation of basal progenitor or stem cells located in the mesenchyme and epithelial layers [2]. While the intrinsic regenerative properties of the epithelium are sufficient to repair mild lesions throughout life, severe injuries such as severe burn, trauma, or inflammation can damage or destroy host basal cells, reducing self-repair capacity below the level necessary to regenerate a functional epithelium [3]. Failure of self-repair leads to incomplete epithelia with damaged physiological functions, raising the risk of complications such as chronic bacterial infection, dehydration, hyperpigmentation, and scar formation.

Severe epithelial injuries are clinically treated by the administration of epithelial tissue grafts as temporary coverage to promote natural healing or as a permanent replacement for severely damaged skin [4]. Epithelial grafts can be divided into two general categories: natural autologous tissue grafts, such as skin flap or buccal mucosa obtained through a surgical procedure; or tissue-engineered composites of biomaterials, which may be seeded with autologous cells harvested through biopsy. The use of both graft types has been found to improve aesthetic and functional outcomes compared to natural healing [5]. However, collection of healthy donor epithelium for tissue grafts is invasive and can involve complications such as discomfort, bleeding, infection, and prolonged healing time. 

Furthermore, there may not be sufficient donor tissue to repair large-scale epithelial injuries. Conversely, while tissue-engineered epithelial substitutes seeded with autologous cells involve minimal secondary injury during the collection of donor cells, questions persist regarding engineered graft safety, efficacy, biocompatibility, availability, and scalability [6]. Particularly, the optimal cell source(s) for cellularized grafts has yet to be determined. Various cells have been studied for epithelial tissue engineering, including somatic cells from the skin, bladder mucosa, buccal mucosa, and adult stem cells (i.e., bone marrow-derived mesenchymal stromal cells [BMSC] [7], or adipose derived stem cells [ASC] [8]). However, these cells exhibit limited self-renewal and expansion capacity, restricted differentiation capacity, and lack telomerase activity. Similar to natural autologous tissues, the invasive collection procedure can be painful, and, in all cases, lengthy culture time is required to expand and characterize cells, diminishing their clinical utility [4].

To address these complications, we have proposed human primary urine-derived stem cells (USC) as an alternative cell source for engineered epithelial tissues [9]. As described below, USC are obtained by urine collection and exhibit chromosomal stability, are not oncogenic, and do not cause hyperpigmentation in vivo. USC possess the capacity to differentiate into diverse cell lineages and form multilayered cell sheets with tight junctions on biomaterial scaffolds, producing a barrier function resembling natural epithelium. Moreover, they secrete abundant paracrine factors that regulate immune reactions, suppress inflammation, promote vascularization and re-epithelization, and induce the migration of host cells. Thus, USC have potential value in epithelial tissue repair and reconstruction.

In this review, we focus on the collection, culture, and characterization of USC and use of USC in the skin and mucosa repair. We also discuss the application of USC in tissue-engineered grafts and by injection of USC-derived paracrine factors. Finally, we address the potential role of USC as a model for drug development and cosmetic product testing.

## 2. Characterization of Urine-Derived Stem Cells

Cells integrated into tissue-engineered epithelial grafts must exhibit a set of properties suitable for epithelial tissue regeneration, including high regenerative capacity, asymmetric division, and large-scale expansion ability. Once implanted, seeded grafts should exhibit long-term self-renewal and tissue repair at the transplant site. From a structural perspective, implanted stem cells should produce stratified layers of differentiated cells connected by tight junctions that develop into an epithelial barrier. Cells must be compatible with the chosen biomatrix, which itself must provide structural support and facilitate the distribution of nutrients and regulatory factors necessary for the growth and differentiation of seeded cells [10].

Though tissue-specific stem cells are primarily associated with organs or tissues, stem cells can also be found in body fluids, such as urine. Indeed, we first demonstrated the presence of USC in human and animal urine [9,11,12]. These cells originate from the parietal cells of the kidney glomeruli [13]. They are distinguished from other urine-derived cells by the ability to attach to culture dishes and proliferate. Being initially oval, they become rice-grain shaped in 3–7 days and exhibit morphological properties of induced lineages when cultured in a differentiation media [14]. They exhibit clonogenicity, as well as a high expansion capacity [14,15]. USC isolated from the upper urinary tract proliferate with a maximum population doubling (PD) of 56.7 and an average doubling time (DT) of 20 h [14]. By optimizing the collection and expansion protocols, 100–140 USC clones can be consistently obtained from each provider over a 24 h urine collection period [15].

Cell surface markers are vital features for stem cell identification. USC stain positive for canonical MSC markers (CD29, CD44, CD73, CD90, CD105), and are negative for hematopoietic lineage and immunogenic markers (CD31, CD34, CD45, CD133, HLA-DR) [9,11,13,14,16,17,18,19]. Apart from the classical MSC markers, USC expresses pericyte markers including CD146, platelet-derived growth factor r beta (PDGF-rβ), and neural/glial antigen 2 (NG2) [9,19,20]. Pluripotent stem cell markers such as octamer-binding transcription factor 3/4 (Oct 3/4), VMyc avian myelocytomatosis viral oncogene homolog (c-Myc), stage-specific embryonic antigen 1/4 (SSEA-1/4), and Kruppel-like factor 4 (Klf-4) have been detected in several studies [9,14,17,19]. Considering the kidney origin, USC is also positive for renal cell markers such as sine oculis homeobox homolog 2 (SIX2), neural cell adhesion molecule (NCAM), and epithelial cell adhesion molecule (ep-CAM), and frizzled class receptor (FZD) [21,22,23]. Up to 75% of USC collected from young- and middle-aged individuals and 57% of USC from seniors (≥50 years old) exhibited telomerase activity (USC-TA^+^) and retained long telomere lengths. Karyotype analysis detected no sign of chromosomal abnormality after serial cultures, and teratomas were not found in vivo [24].

**Figure 1:**
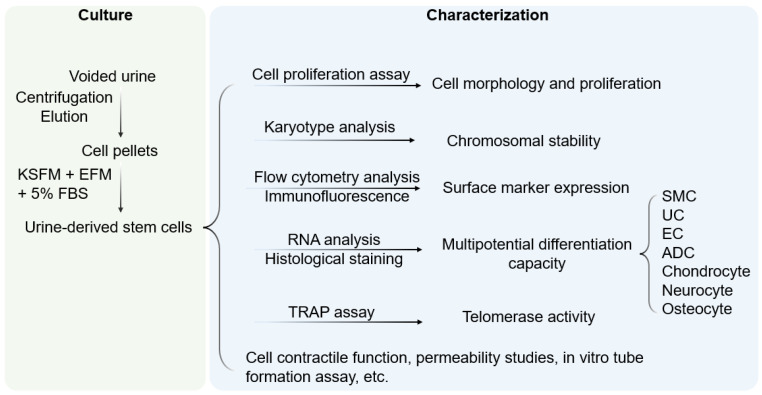
**Culture and characterization of USC.** Voided urine is collected from individuals, followed by centrifugation and elution, after which USC pellets are obtained. Under proper culture conditions, USC becomes rice-grain shaped in 3–7 days, and gradually obtains distinct morphology of induced lineages when cultured in a related differentiation medium. KSFM, embryonic fibroblast medium; EFM, keratinocyte serum-free medium; FBS, fetal bovine serum; SMC, smooth muscle cells; EC, endothelial cells; ADC, adipocytes; TRAP, telomerase repeated amplification protocol.

Evidence from in vitro and in vivo characterization indicated that USC possesses multipotent differentiation capacity [10,13,14,16,25] (Figure 1). In early experiments, USC was observed to have bipotential differentiation ability. When cultured in myogenic and uroepithelial differentiation conditions, they developed morphological and functional properties of smooth muscle cells (SMC) and urothelial cells (UC), including lineage-specific transcripts and proteins such as desmin, myosin, and uroplakins, respectively [14].

However, USC was subsequently shown to give rise to non-urological cell types, such as endothelial, neuronal, osteogenic, chondrogenic, adipogenic, and skeletal myogenic lineages, suggesting a multipotent capacity [13,25]. Indeed, USC have been used in regeneration of multiple tissue types, including bladder [26], urethra [12], kidney [27,28,29], penal tissue [30,31], bone [32,33,34,35], lungs [36], skin [37], nerves [19] and other types of tissue [18,19,34,35,37,38,39,40,41,42,43], as well as in development of preclinical models of tissue repair [17,26,27,28,32,33,34,44,45,46,47,48]. Similar to mesenchymal stem cells, USC exhibits pro-angiogenic and neurogenic paracrine effects [37,49,50]. The immuno-modulatory property of USC is reflected by their ability to inhibit the proliferation of allogeneic lymphocytes and to increase the levels of immunoregulatory cytokines interleukin (IL)-6 and IL-8 in lymphocyte co-cultures [51]. Under hypoxic conditions, USC can improve the secretion of growth factors that promote angiogenesis, re-epithelialization during wound healing [52], and accelerate the repair of injured hepatic tissues [53]. Neovascularization, myogenesis, and innervation can be further enhanced by modification of USC to express the VEGF gene [54].

USC can be non-invasively isolated from voided urine and easily expanded in vitro [13,14,15]. These capabilities offer clear advantages over stem cells from other sources, such as bone marrow or adipose tissue, which require invasive procedures for isolation and expand slowly in the culture [46,55]. Isolation of USC is further enhanced by the lack of tissue dissociation procedures, which increases cell viability and total recovery [14,15]. USC is readily available in voided urine from adults, enabling the collection of 140 USC clones every 24 h [15]. USC has a high self-renewal capacity, possessing telomerase activity and a relatively long telomeres [9], thereby enabling the rapid expansion of isolated cells. At the same time, USC retains chromosomal stability during in vitro culture [9] and is safe to use in vivo without any risk of oncogenicity [10,11,12,13,14,15,16,19,23,25,26,31,32,34,35,37,54,56]. USC exhibits multi-lineage differentiation capacity and improved differentiation of urothelial and endothelial cells compared to BMSC, ASC, or ESC/iPSC [10,25,46]. Furthermore, because of their autologous origin, implantation of USC in vivo is unlikely to elicit a rejection reaction. Collectively, USC is a technically facile, autologous stem cell source with versatile therapeutic potential.

USC is originally from a single cell clone, offering pure renal stem cells at the beginning (*p*0). Subsequently, some USC starts gradually differentiating with heterogeneous at a small cell population with passaging. About 50–75 of the USC population retain telomerase activities at the early stage of culture (<*p*5) [57], indicating these USC remaining stemness [13,14,15,22,58] Thus, it is recommended to use USC in the early passage (<*p*5) for cell therapy to better promote epithelial tissue repair and the wound healing.

## 3. Reconstruction of 3D Epithelial Tissue

Epithelial tissues, despite their diverse locations in the human body, share some universal qualities in structure and function. The epithelium exhibits stratified architecture with a multilayer of superficial cells, intermedium cells, and basal cells with abundant tight junctions, located within an extracellular matrix (ECM) that strengthens vertical and longitudinal cell–cell interactions and cell–ECM adhesion. The epithelium is supported and nourished by underlying connective tissue containing blood vessels formed with endothelial cells and stromal cells. 

Thus, to regenerate these complex physiological structures, engineered grafts should contain epithelial, endothelial, and stromal cells within a matrix that supports the formation of an integrated structure, long-term survival, and recapitulation of basic epithelial functions [2,59]. Additional cell types may be included in tissue-engineered grafts to fulfill specialized functional requirements; for instance, the inclusion of hair bulbs to promote hair growth [60]. USC have been found to have numerous properties that enhance the repair and regeneration of diverse epithelial tissues, particularly skin and urogenital mucosa.

## 4. Methods to Enhance Skin Regeneration Using USC

Traumatic and chronic wounds that damage the skin or other epithelial tissues affect nearly 2% of the total population in the United States. While traumatic wounds are typically caused by mechanical, chemical, or thermal insult, chronic non-healing wounds refer to injuries that remain unhealed for more than one month, most commonly because of abnormal repair processes. Patients with chronic wounds suffer from decreased quality of life and elevated risk of infection, sepsis, or death due to impaired skin barrier function [3]. According to an analysis published in 2021, total U.S. healthcare expenditures for all wounds ranged from USD 28.1 billion to USD 96.8 billion per year. Despite the high prevalence, mortality, and healthcare spending, funding for relevant studies of chronic wounds remains low in the United State. At the same time, the wound care market is rapidly growing in countries around the world, reaching an estimated USD 4 billion in China by 2027 [61].

Skin is the largest organ, covering the external surface of the human body, and has several critical biological functions, including protection, sensory transduction, thermoregulation, metabolic regulation, and sexual signaling. It is composed of three main layers: epidermis, dermis, and hypodermis. The epidermis contains abundant keratinocytes, which form four epithelial layers with distinctly different structures and functions. The superficial layer stratum corneum consists of dead, anucleate keratinocytes for protection and prevention of dehydration. The stratum granulosum contains flattened keratinocytes undergoing keratinization for blockage of harmful substances. The thickest layer is the stratum spinosum, responsible for keratin synthesis. Finally, the basal layer stratum contains progenitor cells with strong mitotic activity for the epidermal tissue regeneration [4,62].

Other cell types in the epidermis include melanocytes, Langerhans cells, and Merkel cells, which provide melanin production, immune defense, and sensation, respectively. The dermis is a connective tissue layer underlying the epidermis which acts as a supportive and nutritive layer. The dermis is divided into a loose papillary layer with dermal papillae projecting into the epidermis, strengthening the connection between dermis and epidermis, and a thicker underlying reticular layer rich in fibers to provide elasticity. Fibroblasts are the major cell type in the dermis. The dermis is richly innervated with sensory and effective neurons and contains vascular plexus and lymphatic vessels as well as several skin appendages such as hair follicles, sweat, and sebaceous glands. The hypodermis consists of loose adipose connective tissue, which provides nutrients and oxygen and connects the upper skin layers to deep tissues [2].

### 4.1. Skin Regeneration Requirements

This complex structure consisting of several main layers and sublayers with special appendages makes the development of a tissue-engineered skin graft a challenging task. *A priori*, a tissue-engineered skin substitute must possess several properties that mimic natural skin. At a minimum, the graft should include an epidermal layer and a dermal layer as a bed to support and nourish the epidermis. Cells in these layers should be able to attach, proliferate, differentiate, establish connections with surrounding tissues and eventually form elastic structures adaptable to irregular forces. As a protective barrier, the substitute should have a robust barrier function, protecting underlying tissues from dehydration, mechanical stimulation, and microbial infection. Low immunogenicity and the capacity to endure a hypoxic environment are important for the long-term success of skin substitutes, suggesting autologous cells are a more ideal cell source than allogeneic cells. Finally, the production of skin substitutes with long shelf lives and simple and non-invasive donor collection protocols will reduce cost and improve patient acceptance [63].

### 4.2. Skin Cell-Sheet

The optimal skin substitutes for cutaneous repair involve cell sheets formed of one or more layers of compactly aggregated cells of multiple types with natural ECM, needing no artificial scaffolds. Because an intact tier of cell sheets can be detached from the culture dish through mechanical manipulation, digestive enzymes are not required, thereby preserving cell surface proteins and junctions without reducing the cell viability [64]. The strong adhesive properties, mechanical strengths resembling natural tissue, and ability of skin cell sheets to develop into stratified structures offer clear advantages for epithelial tissue regeneration [65,66]. Cell sheets have been evaluated in cornea, bladder, cardiac defect, and skin wound repair [64].

Cerqueira et al. developed 3D constructs using cell sheets with multiple cell types, including keratinocytes, fibroblasts, and endothelial cells, which promoted neo-vessel formation and re-epithelization after transplantation to skin wound of mice model [64]. Relevant to this review, two studies recently reported that cell sheets of USC enhanced rotator cuff healing in a canine model [67] and increased osteogenic and cementogenic protein expression in osteogenic matrix PDLSC sheets containing human periodontal ligament stem cells and USC at a 1:2 ratio [68]. These studies suggest skin sheets comprised of USC will be useful for various epithelial tissue regeneration, including skin, urothelium, and cornea.

### 4.3. Cell-Seeded Biomatrix for Epidermal Tissue Reconstruction

The successful application of biomatrix seeded with cells or cell products [69,70,71] as epidermal grafts for skin wound treatment has led to an expanded interest in USC as a cell source for tissue engineering (Table 1). Wounds treated with USC-seeded polycaprolactone/gelatin nanofibrous membranes showed enhanced wound contraction and improved re-epithelialization versus scaffold alone, resulting in faster wound closure [37]. Experimental analyses revealed that regenerated skin tissue contained a thicker granular layer, more collagen production, increased angiogenesis, and cutaneous appendages structurally similar to sebaceous glands and hair follicles. Similar results were obtained when human USC were incorporated with surface-structured bacterial cellulose scaffold as a composite for the repair of a full-skin defect of a rabbit model [50]. In a mouse wound model, Zhang et al. [52] preconditioned USC-seeded small intestinal submucosa (USC-SIS) graft with hypoxia culture media and subsequently observed faster wound healing with better skin regeneration than in a normoxia environment, indicating hypoxic pretreatment to be a simple method for enhancing skin regeneration.

Nerve fibers exist in the dermis and stretch into the epidermis, playing a vital role in detecting outside stimuli and hair movement. In patients suffering burn injuries or diabetic ulcers, the sense of pain, temperature, and touch is severely damaged, reducing the quality of life. Thus, facilitating neural re-innervation is critically important in tissue-engineered skin construction. Several autologous stem cells including skin-derived precursor cells (SKP) and ASC in the hypodermis, present some potential to differentiate into Schwann cells [72]. However, collection of SKP or ASC requires skin biopsy or liposuction. The neurogenic-lineage differentiation capacity of USC was researched by several teams [19,73,74]. Guan et al. injected undifferentiated USC into damaged rat brain and found they survived, migrated to other sites of the brain, and expressed neuronal lineage-specific proteins, such as nestin, b-III-tubulin, and glial fibrillary acidic protein (GFAP).

**Figure 2:**
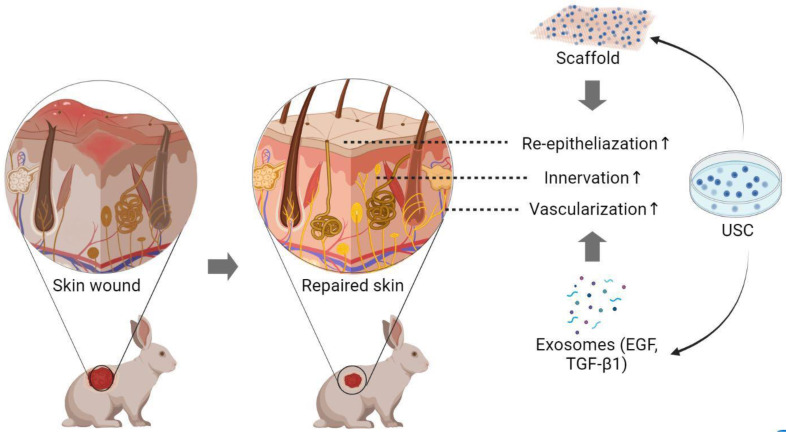
**Applications of USC in skin regeneration and wound repair**. When seeded on the scaffold and transplanted to a skin wound, USC promotes angiogenesis, re-epithelization, and innervation by generating various exosomes transporting growth factors such as VEGF and TGF-β1. When embedded in hydrogel and injected into the brain of a rodent, USC presents the differential potential for versatile neurocytes. USC, urine-derived stem cells; VEGF, vascular endothelial growth factor; TGF-β1, transforming growth factor β1; ↑ indicating increase. Created with BioRender.com (accessed on 27 July 2022).

In 2017, Liu et al. [73] produced iPSC by reprogramming patient USC with non-integrating Sendai viral vectors. The addition of a neural induction tissue culture medium elicited markers consistent with neural progenitor cells, including A2B5, SOX1, PAX6, and nestin. Upon transplantation into mice with spinal cord injury, induced neural progenitor cells integrated into the existing spinal tissue and expressed markers of astrocytes, neurons, and axons after 8 weeks. However, cutaneous studies indicate USC enhances innervation primarily by inducing the in-growth of surrounding nerve tissues via neurogenic paracrine effects, rather than differentiating into nerve tissue themselves [49]. While these data indicate USC can differentiate into several skin cell types and promote re-epithelization, re-innervation, and angiogenesis after full-thickness skin defects [37] (Figure 2), additional characterization is required to understand the ability of USC-seeded, tissue-engineered skin constructs to improve clinical outcomes for severe skin injuries.

**Table 1:** Application of various autologous stem cells in wound treatment.

Cell Type	Wound Treatment	Outcomes
**BMSC**		
*Animal expt.*	Diabetic/excisional wounds *, **, *** Skin burns ** Radiation burns/dermatitis *, **	M1 macrophages ↓, M2 macrophages ↑ [75]; Re-epithelialization, granulation tissue, angiogenesis, collagen deposition ↑; inflammation ↓ [76,77,78]; MMP1 expression, pro-collagen ↑ [79]
*Clin trials*	Diabetic foot ulcers *, fibrin spray, *** Bullosis diabeticorum *	Ulcer size, healing time, complications ↓, vascularity ↑ [80,81,82]; clinical outcomes ↑, prevent lower limb amputation [83]
**AT-MSC**		
*Animal expt.*	Diabetic wounds **, *** Excisional wounds *** Radiation burns *	Innervation, M2 macrophages, granulation tissue, angiogenesis ↑; Inflammation ↓ [8,84]; Wound closure, epidermal/dermal structure ↑ [85,86]
*Clin trials*	Ablative laser: niacinamide cream Low extremity ulcers ***	MMP-1 and MMP-2 expression ↓, type 1 collagen expression ↑ [87]; dermal angiogenesis, wound closure ↑; complete closure time ↓ [88,89]
**UC-MSC**		
*Animal expt.*	Diabetic wounds *, *** Burn wounds * Excisional wounds **	Wound closure, re-epithelialization, angiogenesis ↑ [90,91]; inflammation ↓ [92]; fibroblasts-myofibroblasts transition ↓ [89,90,91,92]
*Clin trials*	Diabetic foot ulcers * Cesarean section skin scars: patch Ablative laser: serum and cream Epidermolysis bullosa *	Ulcer healing ↑ [93]; no evident enhancement in skin repair [94]; post-treatment erythema ↓, recovery time ↓ [95]; M2 macrophage polarization ↑; mast cell infiltration, pain ↓ [96]
**WJ-MSC**		
*Animal expt.*	Excisional wounds * Atopic dermatitis *	No evident enhancement [97,98] Epidermal thickness, inflammation ↓ [99]
*Clin trials*	Diabetic foot ulcers ***	Wound size, healing time ↓ [100]
**ASC**		
*Animal expt.*	Pressure sores * Excisional wounds **	Wound healing ↑ [101]; dermal fibroblasts migration and proliferation, wound closure ↑ [102]
**UCB-MSC**		
*Animal expt.*	Diabetic wounds *, *** Excisional wounds *	Wound healing, angiogenesis ↑ [103,104,105]
**USC**		
*Animal expt.*	Excisional wounds *, *** Diabetic wounds **	Wound closure, re-epithelialization, angiogenesis, collagen deposition ↑ [37,49,50,52]

Abbreviations: Clin, clinical; expt., experiment; MSC, mesenchymal stem/stromal cell; BMSC, bone marrow derived stromal cells; ASC, adipose-derived stem cells; UC, umbilical cord; AF, amniotic fluid; UCB, umbilical cord blood; WJ, Wharton’s jelly; USC, urine-derived stem cell; * indicating cell therapy; ** indicating exosome therapy; *** indicating scaffold implantation; ↑ indicating increase; ↓indicating decrease.

## 5. The Role of USC in Urothelial Mucosa Repair

The urethra is comprised of two primary tissues: the mucosa and the urothelium. The urothelium covers the inner surface of the urethra and urinary bladder and is a slow-cycling epithelial tissue composed of stratified layers, including a superficial layer of umbrella cells for protection against urine, an intermediate layer, and a layer of basal cells with a high self-regeneration capacity [66]. Urethral injury is a common result of a variety of conditions such as urethral stones, abscesses, hypospadias, major trauma, and chronic inflammation. The urethral injury occurs in approximately 10% of male patients who suffer blunt or penetrating trauma and 6% of females following fractures of the pelvic [106]. Urethral injury leads to long-term debilitating outcomes such as urinary incontinence and dysuria [107]. Clinical repair of severe ureteral injuries involves urethroplasty with mucosal transplants to restore urinary tract function [5], illustrating the critical need for improved regenerative strategies.

### 5.1. Urothelial Regeneration Requirements

**Figure 3:**
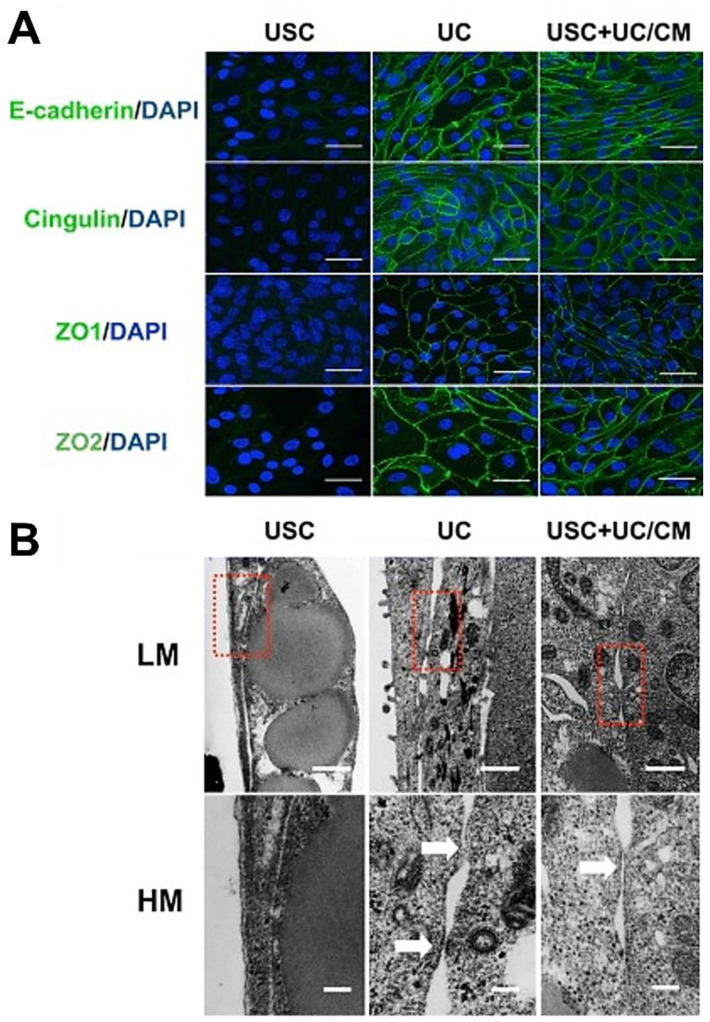
**The formation of cellular junctions between urothelial induced USC.** (**A**) Images of immunofluorescent staining indicated the comparable formation of tight junctions with stained markers (E-cadherin, Cingulin, ZO1, and ZO2) in both UC and urothelial differentiated USC, but not in USC alone. Scale bar = 40 μM. (**B**) Tight junctions and desmosomes were observed in the intercellular space of both UC and urothelial differentiated USC, whereas not found in USC alone through TEM. The red dashed box and white arrows indicate tight junctions and desmosomes. LM scale bar = 500 nm, HM scale bar = 100 nm. Abbreviations: TEM, transmission electron microscopy; USC, urine-derived stem cells; UC, urothelial cells; UC/CM, urothelium conditioned medium. Images are adapted from *Stem Cell Res Ther.* [56] with permission. (License link: http://creativecommons.org/licenses/by/4.0/ accessed on 13 April 2022).

**Figure 4:**
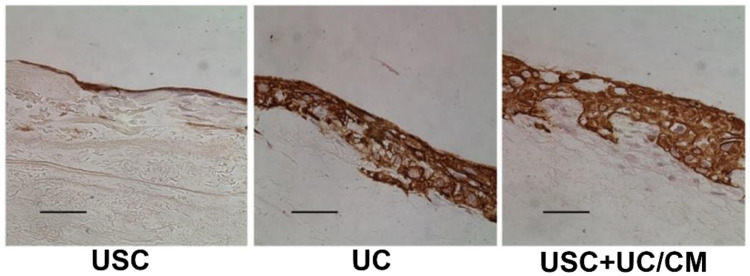
**Multiple-layered structure of the USC cell sheet on SIS.** Treated with UC/CM, USC formed a cell sheet with five to seven layers resembling natural urothelium, while USC alone generated a thin layer of urothelium. Scale bar = 50 μm. Abbreviations: USC, urine-derived stem cells; UC/CM, urothelium conditioned medium; SIS, small intestinal submucosa. The images are adapted from *Stem Cell Res Ther.* [56] with permission. (License link: http://creativecommons.org/licenses/by/4.0/ accessed on 13 April 2022).

**Figure 5:**
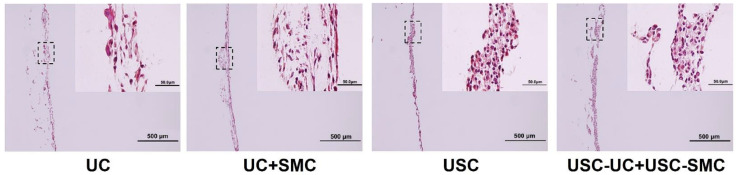
**Cell sheets formed under 3D dynamic culture.** The co-culture of USC-induced UC and SMC under 3D dynamic conditions gave rise to cell sheets with up to 14 layers and denser structure, compared with USC or UC alone, assessed by Masson’s trichrome staining. Abbreviations: USC, urine-derived stem cells; UC, urothelial cell; SMC, smooth muscle cell. The images are adapted from *Biomaterials* [16] with permission. (License number: 5292510480483).

**Figure 6:**
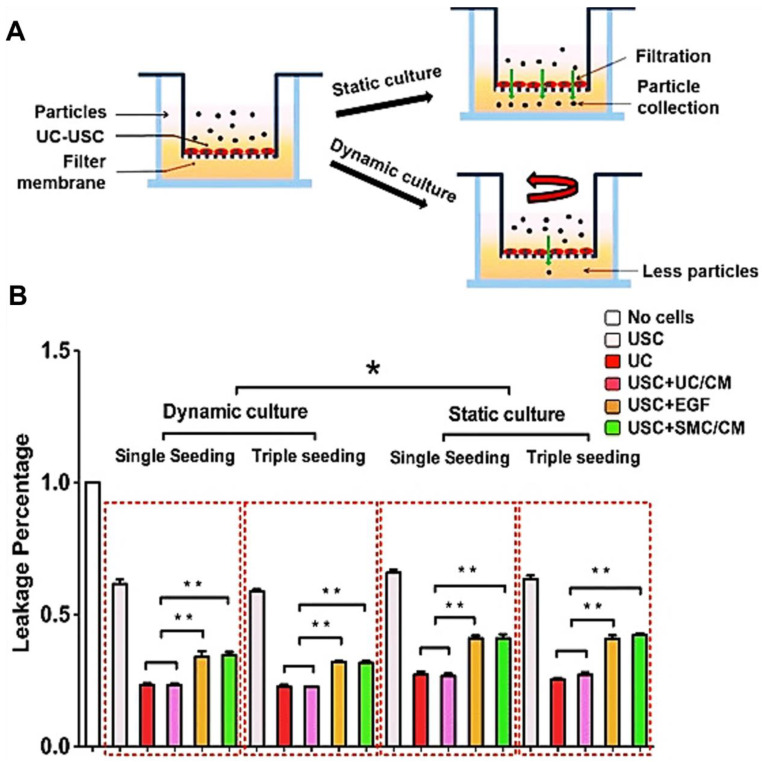
**Permeability barrier function of urothelial induced USC.** (**A**) Permeability test in vitro showed fewer leaking particles passed through urothelial induced USC formed barrier under dynamic culture, compared with static culture. (**B**) The qualification of leakage percentage indicated enhanced differentiation and barrier function of urothelial induced USC under UC/CM and dynamic culture, resembling physical urothelium. Abbreviations: USC, urine-derived stem cells; UC, urothelial cells; SMC, smooth muscle cells; CM, conditioned medium; UC/CM, urothelium-conditioned medium; SMC/CM, smooth muscle cell-conditioned medium; EGF, epidermal growth factor; single seeding, seeding cells only once; triple seeding, seeding cells each at first 3 days. * *p* < 0.05, ** *p* < 0.01. Images are adapted from *Stem Cell Res Ther.* [56] with permission. (License link: http://creativecommons.org/licenses/by/4.0/ accessed on 13 April 2022).

Regeneration of the bladder wall or urethral tissue for lower urinary tract reconstruction requires stratified epithelial layers with barrier functions, stromal cells for support, and endothelial cells for rapid angiogenesis [107], all seeded on the scaffold that supports multiple layers of urothelium with smooth muscle cells generated in vitro. Existing studies we conducted demonstrated that urothelial cell sheets with barrier function and 3D constructs of urothelial cells co-cultured with smooth muscle cells on scaffolds have good therapeutic potential for regeneration of urological tissues [56] (Figure 3, Figure 4, Figure 5 and Figure 6). In cell source collection, autologous UC, SMC, and endothelial cells are often obtained via tissue biopsy or invasive procedures [108,109].

To optimize the regeneration of the urothelial tissues, a favorable cell source and scaffold has to be carefully selected. Various cell types have been evaluated for urological tissue engineering, including autologous UC [7,65,66,110,111,112,113], epithelial cells originating from buccal mucosa [114], embryonic stem cells (ESC) [40], induced pluripotent stem cell (iPSC) [40], BMSC [7], and adipose-derived stem cells (ASC) [115]. However, obtaining these cells requires either invasive procedures (i.e., BMSC) or costly methods (i.e., iPSC). Thus, an easily assessable and largely expandable stem cell source is needed. Similarly, various options have been developed for the scaffold materials, including porcine xenografts, manufactured natural scaffolds, amniotic membranes, and synthetic and 3D bio-printed scaffolds [6]. Ideal scaffolds should have porous microstructures but the best combination of structural integrity, integration with preferred cell types, and functional development of physiological normal tissues.

### 5.2. Implantation of USC with Scaffolds for Urogenital Repair

**Figure 7:**
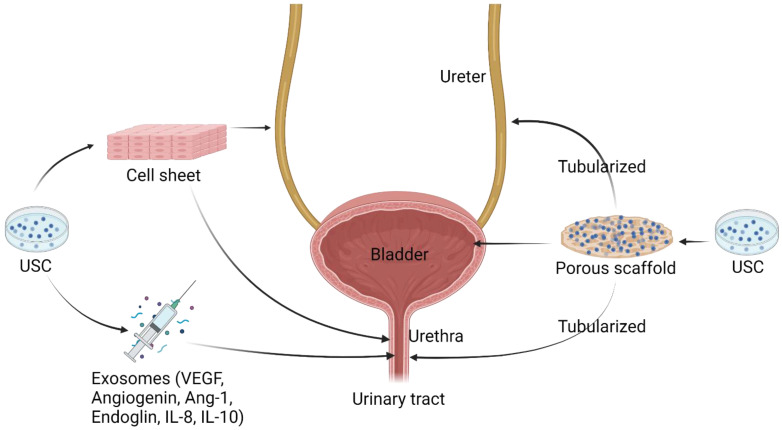
**Applications of USC in urothelial mucosa reconstruction.** The combination of USC and flat or suberized biomaterial scaffolds as implantation has been investigated in bladder repair, ureter, and urethra reconstruction. The injection of growth and immunoregulatory factors secreted from USC shows effectiveness in the treatment of urethral stricture. USC, urine-derived stem cell; VEGF, vascular endothelial growth factor; Ang, angiogenin; IL, interleukin. Created with BioRender.com accessed on 27 July 2022.

We have reported human and animal USC can differentiate into UC, SMC, endothelial cells, and interstitial cells, suggesting USC is a potential cell source for tissue-engineered bladder or urethra tissue for the urinary tract repair [9,11,14,56] (Figure 7). Bodin et al. [16] constructed a tissue-engineered urinary conduit using a porous bacterial cellulose scaffold seeded with USC obtained from the upper urinary tract. USC differentiated into SMC and UC in an inductive medium and formed multilayer urothelium after co-culture on the conduit under rotational culture conditions, producing more cell layers with improved cell infiltration versus normal human SMC and UC in vivo.

The utilization of USC from the upper urinary tract is especially valuable for patients who underwent cystectomy or those with nephrostomy tubes. To address concerns that synthetic scaffolds might inhibit the diffusion of molecules important for tissue repair, Liu et al. [12] used preconditioned 3D porous small intestinal submucosa (SIS) instead of bioengineered scaffolds as the carrier for USC. USC-derived UC and SMC formed multilayered structures on SIS, and the graft was implanted in the urethral mucosa defect site of the rabbit. Three months after implantation, intact urothelium formed at the defect, and epidermal cells covered the surface. Moreover, unlike the group using SIS alone, minimal or no inflammation, fibrosis, or urethral strictures were observed in the USC-seeded SIS group.

Patients who underwent gastrocystoplasty commonly suffer from postoperative complications such as dysuria–hematuria syndrome and gastrointestinal mucosa metaplasia [111]. In theory, augmentation of the stomach flap with urothelium on the surface could enhance barrier function and prevent urine leakage, thus improving clinical outcomes. Zhang et al. [111] seeded canine SMC and UC on SIS to create multilayered urothelium and combined it with a de-mucosalized stomach patch. The gastric graft was fully covered by regenerated stratified urothelium and showed reduced metaplasia and calcification compared to the de-mucosalized gastric patch alone. Graft shrinkage was significantly improved when co-administered with BOTOX^®^. Lee et al. [26] further demonstrated the use of USC in bladder tissue engineering. Combining USC with heparin-immobilized bFGF-loaded scaffold improved compliance and capacity of rat bladder within 8 weeks. Histological analysis showed that the myogenic and urothelial differentiated USC seeded on this modified scaffold gave rise to thicker smooth muscle and urothelium with more compact submucosa tissue compared with the scaffold alone.

## 6. Applications of USC-Derived Exosomes

**Figure 8:**
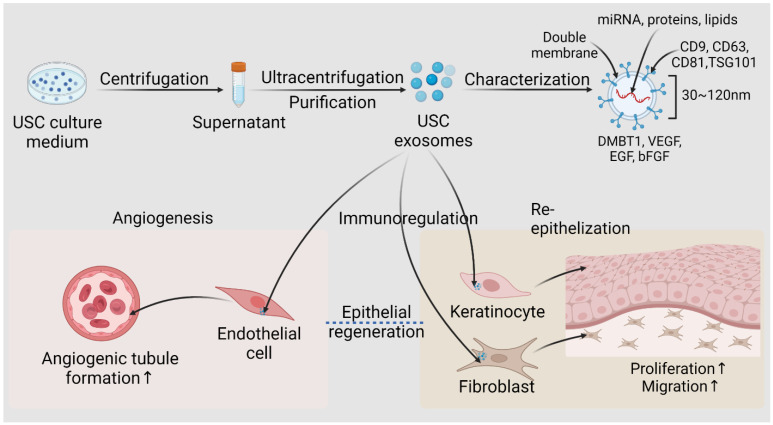
**Isolation, characterization, and roles of USC-exosomes in epithelial regeneration.** USC generates abundant exosomes, which are 30–120 nm-sized vesicles with lipid bilayer membrane harboring molecules such as miRNA, protein, and lipids. Exosomes are secreted extracellularly and exert angiogenic, immunoregulatory, or trophic effects on the surrounding microenvironments. Urine-derived stem cell; DMBT1, deleted in malignant brain tumors 1; VEGF, vascular endothelial growth factor; TGF-β1, transforming growth factor-β1; EGF, epidermal growth factor; bFGF, basic fibroblast growth factor; ↑ indicating increase. Created with BioRender.com accessed on 27 July 2022.

Exosomes are complex vesicles produced and secreted by various cells that mediate cell–cell interactions in many physiological processes, including inflammation, trauma, and sepsis [116]. The effectiveness of exosomes secreted by USC in promoting wound healing has been demonstrated in numerous tissues and for diverse injuries and diseases [50,52] (Figure 8). Likewise, USC produce exosomes containing exosomal markers CD63 and TSG101 and proteins that promote angiogenesis, ECM organization, and epithelial cell proliferation [49]. Indeed, in vitro and in vivo experiments demonstrate USC secretomes contain a rich variety of growth and immunoregulatory factors that have proangiogenic and immunoregulatory effects on a wide range of disease conditions, including skin and urogenital diseases [9,25,31,54]. It is of particular interest for skin regenerative studies that USC-derived exosomes contain large quantities of deleted in malignant brain tumors 1 (DMBT1), which is a pro-regenerative protein that regulates VEGF-A. Accordingly, USC-derived exosomes stimulate blood vessel formation, promote re-epithelialization and enhance angiogenesis when administered to diabetic ulcers in mice [49].

In addition, USC releases myogenic and endothelial growth factors such as Angiogenin, Ang-1, and Endoglin, which may act through paracrine mechanisms to regulate USC differentiation while also stimulating the regeneration of adjacent tissues, ultimately promoting appropriate neovascularization, myogenesis, and neuronal innervation [25,31,54] in the treatment of urinary incontinence [117]. When applied to diabetic rodents with different human-induced diseases, USC exosomes can elicit disease-specific regenerative responses, including elevating smooth muscle/total collagen ratio after intra-cavernous injection in erectile dysfunction rat models [30], preventing podocyte apoptosis and reducing caspase-3 expression in diabetic nephropathy [28,118] and improving skin cell migration and proliferation of diabetic wounds [49]. USC-secreted immunomodulatory factors such as IL-8, and IL-10 may also contribute to the absence of the rejection of human USC in immunocompetent rodents without an immunosuppressive treatment [30], further broadening their potential use for xenogeneic transplantation.

## 7. Conclusions and Future Directions

The application of USC in epithelial tissue repair is versatile, ranging from a cell-seeded scaffold, and cell sheet to cell and exosome injection therapy (Table 2). In general, USC exerts regenerative capacity by secreting paracrine factors, recruiting neighboring cells, and differentiation towards specific cells. However, their role in skin and urothelium repair is slightly different, according to existing studies. USC primarily promotes skin regeneration by releasing trophic and immunoregulatory factors to stimulate the crawling of resident cells and ultimately accelerate the re-epithelization, neovascularization, and innervation. While in urothelial tissue regeneration, apart from the paracrine effect, USC can be easily induced and differentiate into urothelial cells and smooth muscle cells due to their kidney origin, participating in the reconstruction of the urogenital tract by forming multi-layered urothelium with tight junctions and barrier functions.

Indeed, there are limitations in current studies to be solved. For instance, when collected from females or children, the contamination of urine samples by bacteria from skin, colon, or vaginal secretion occurs frequently. The collection of urine demands amelioration. In addition, despite abundant previously performed in vitro and in vivo experiments, no clinical trials using USC in the actual treatment of patients are found. Autologous USC might also act as a rich and promising source of stem cells for the treatment of ISD in elder women with SUI [117].

As an easily accessible cell source, human USC offers clear advantages over stem cells from other sources for tissue regeneration and wound healing. USC does not require tissue dissociation procedures with digestive enzymes, thus better preserving cell viability. USC possesses robust regeneration capacity without exhibiting oncogenic potential or chromosomal aberrations. Greater than 1 × 10^8^ cells can be produced for cell implantation within three weeks after 24 h collection of urine. USC exerts immune-regulatory or trophic effects via the secretion of several paracrine factors, accelerating angiogenesis, innervation, and re-epithelialization. Tight junction and barrier function are well developed in multiple layers of USC-seeded structures for urothelial tissue reconstruction and skin wound healing. The distinctive advantages of human USC offer potential benefits for epithelial tissue reconstruction, disease modeling, and drug development as listed below:

- Transplantation of USC cell sheets or USC-seeded scaffolds has potential in the treatment of genitourinary defects or wound repair for patients with a diabetic ulcer or burn injury. Their exosomes can be extracted and manufactured as commercial products for skin wound healing. More studies on optimization of USC therapy are needed before clinical trials start using patients’ USC for skin repair and wound healing are needed in the near future.
- Autologous USC is an optimal stem cell source for the treatment of other urological disorders, such as intrinsic urethral sphincter deficiency in elder women with stress urinary inconstancy, erectile dysfunction, and renal insufficiency. USC-hydrogel will be an easily injectable, variable, degradable material with high biocompatibility and biosafety for cell therapy.
- USC displays distinct values in personalized disease modeling or biomarkers, including renal tumors [119], diabetic nephropathy [120], or various nephritis.
- Three-dimensional cultures of USC including organoids offers alternative approaches for the assessment of renal toxicity or mitochondrial toxicity [121,122,123], and cosmetic care production testing [124].

## Figures and Tables

**Table 2:** Applications of USC in epithelial tissue repair.

Epithelial Tissues	Biomaterial	Key Outcomes
Cell-seeded scaffold for urinary tract reconstruction in vivo	3D porous SIS Porous bacterial cellulose scaffoldComposite scaffold	Urethral caliber, SM content, vessel density ↑ [12] 3D multilayered urothelium, cell matrix infiltration [10,16] Bladder capacity, compliance, SM content, multi-layered urothelium, submucosa layers ↑ [26]
In vitro cell sheet structure	3D porous SIS	A multilayered urothelium with barrier function [56]
Exosome therapy for urinary tract reconstruction in a rodent model	Collagen-I gel	Angiogenesis, in vivo cell survival, myogenic, and nerve regeneration ↑ [25]
Cell therapy for SUI in rodent models	Collagen hydrogel Alginate microbeads Collagen-I gel	Cell survival, cell recruitment, myogenesis, innervation, neovascularization ↑ [54]
Exosome therapy for ED in diabetic rat model	PBS	Endothelial functional protein eNOS and CD31 expression, ICP ↑ [30]
Cell seeded scaffold and exosome therapy for skin defect repair in vivo	Polycaprolactone/gelatin nanofibrous membranes, surface-structured bacterial cellulose, 3D porous SIS	Wound closure rate, re-epithelialization, and angiogenesis ↑ [37,50,52]
Exosome therapy for diabetic ulcer in rodent model	PBS	Wound closure rate, re-epithelialization, skin cell proliferation ↑; Scar formation ↓ [49]

Abbreviations: NM, not mentioned; SUI, stress urinary incontinence; SM, smooth muscle; SMC, smooth muscle cell; BSM, bladder submucosa scaffold; SIS, small intestinal submucosa; ED, erectile dysfunction; VEGF, vascular endothelial growth cell; EC, endothelial cell; PBS, phosphate buffer solution; FGF, fibroblast growth factor; PDGF, platelet-derived growth factor; EGF, endothelial growth factor; UC, urothelial cell; TGF, transforming growth factor; ICP, intracavernous pressure; eNOS, endothelial nitric oxide synthase; ↑ indicating increase; ↓indicating decrease.
